# CRABP2 regulates infiltration of cancer-associated fibroblasts and immune response in melanoma

**DOI:** 10.32604/or.2023.042345

**Published:** 2023-12-28

**Authors:** SHUANGSHUANG ZENG, XI CHEN, QIAOLI YI, ABHIMANYU THAKUR, HUI YANG, YUANLIANG YAN, SHAO LIU

**Affiliations:** 1 Department of Pharmacy, Xiangya Hospital, Central South University, Changsha, 410008, China; 2 National Clinical Research Center for Geriatric Disorders, Xiangya Hospital, Central South University, Changsha, 410008, China; 3 Pritzker School of Molecular Engineering, Ben May Department for Cancer Research, University of Chicago, Chicago, IL, USA; 4 Department of Pathology, The Second Affiliated Hospital of Shandong First Medical University, Taian, 271000, China

**Keywords:** CRABP2, Melanoma, PD-1, Cancer-associated fibroblasts, Immune infiltration

## Abstract

Finding biomarkers for immunotherapy is an urgent issue in cancer treatment. Cellular retinoic acid-binding protein 2 (CRABP2) is a controversial factor in the occurrence and development of human tumors. However, there is limited research on the relationship between CRABP2 and immunotherapy response. This study found that negative correlations of CRABP2 and immune checkpoint markers (PD-1, PD-L1, and CTLA-4) were observed in breast invasive carcinoma (BRCA), skin cutaneous melanoma (SKCM), stomach adenocarcinoma (STAD) and testicular germ cell tumors (TGCT). In particular, in SKCM patients who were treated with PD-1 inhibitors, high levels of CRABP2 predicted poor prognosis. Additionally, CRABP2 expression was elevated in cancer-associated fibroblasts (CAFs) at the single-cell level. The expression of CRABP2 was positively correlated with markers of CAFs, such as MFAP5, PDPN, ITGA11, PDGFRα/β and THY1 in SKCM. To validate the tumor-promoting effect of CRABP2 *in vivo*, SKCM xenograft mice models with CRABP2 overexpression have been constructed. These models showed an increase in tumor weight and volume. Enrichment analysis indicated that CRABP2 may be involved in immune-related pathways of SKCM, such as extracellular matrix (ECM) receptor interaction and epithelial-mesenchymal transition (EMT). The study suggests that CRABP2 may regulate immunotherapy in SKCM patients by influencing infiltration of CAFs. In conclusion, this study provides new insights into the role of CRABP2 in immunotherapy response. The findings suggest that CRABP2 may be a promising biomarker for PD-1 inhibitors in SKCM patients. Further research is needed to confirm these findings and to explore the clinical implications of CRABP2 in immunotherapy.

## Introduction

Immunotherapy, basing on the principle of overcoming tumor immune escape and boosting the immune response, brings comprehensive innovation to the field of tumor therapy [1,2]. The most representative category of immunotherapy is immune checkpoint inhibitors (ICIs), which have become the most widely used immunotherapy in clinical practice [3,4]. Abnormal upregulation of immune checkpoint molecules in tumors inhibits T cell signaling, while ICIs acts as monoclonal antibodies to obstruct above pathway and resume T-cell-mediated recognition and clearance for tumor cells [5]. The targets of ICIs are concentrated in cytotoxic T lymphocyte-associated antigen-4 (CTLA-4), programmed cell death-1 (PD-1), and programmed death ligand-1 (PD-L1) [6]. CTLA-4 is expressed on T cells and helps to regulate the immune response by sending a signal to T cells to stop attacking cells that are not infected with a virus or bacteria [7]. PD-1 is expressed on T cells and helps to regulate the immune response by sending a signal to T cells to stop attacking cells that are already infected with a virus or bacteria [8]. PD-L1 is expressed on tumor cells, and it can bind to PD-1 on T cells, which sends a signal to the T cell to stop attacking the tumor cell [9]. Despite the revolutionary success of immunotherapy in tumor treatment, some patients still exhibit innate or acquired resistance to ICIs, exhibiting no response or even resistance [10]. Therefore, finding biomarkers to distinguish between patients with and without response to ICIs, thereby improving the clinical response rate of ICIs, is an urgent and important issue.

Melanoma is one of the most common malignant tumors, which is highly invasive and tends to metastasize early [11]. The response of melanoma to chemotherapy has been generally poor for a long time, while the introduction of immunotherapy and targeted therapy in the past decade has completely changed the treatment situation of melanoma [12]. Following the first clinically used ICI, the CTLA-4 inhibitor ipilimumab, has been approved for the treatment of advanced melanoma, PD-1 inhibitors and PD-L1 inhibitors have also been shown to significantly prolong survival of melanoma [13,14]. Although the successful clinical application of numerous ICIs has completely changed the treatment of melanoma, only about half of the patients showed lasting clinical benefits [15]. For patients with melanoma, reliable biomarkers for predicting response to immunotherapy and new targets for overcoming drug resistance are still urgently needed to overcome immune resistance more effectively.

The tumor microenvironment (TME) has been confirmed to be closely related to the occurrence, progression and metastasis of tumors, significantly affecting the diagnosis, survival outcome and sensitivity to clinical treatment of tumors [16]. Stromal cells in TME, namely cancer-associated fibroblasts (CAFs), are the core role of TME in solid tumors and can secrete a variety of cytokines and metabolites, thus promoting tumor cell proliferation, angiogenesis, invasion, metastasis, and extracellular matrix (ECM) remodeling [17–19]. In addition, CAFs affects susceptibility of tumor to chemotherapy and immunotherapy by forming osmotic barriers and regulating immune responses [20]. CAFs influence the antitumor activity of T cells and promote immune escape by inducing the overexpression of ligands in immune checkpoint molecules such as PD-L1 and PD-L2 in its own cells and other cells in TME [21]. Some specific CAFs subtypes have also been found to express inflammatory factors and immunosuppressive factors as IL-33 and CXCL14, and affect the ICI efficacy in melanoma, renal cancer, and bladder cancer [22].

Cellular retinoic acid-binding protein 2 (CRABP2) is a significant part of the retinoic acid (RA) signaling pathway, and its function is mainly to regulate the transcription of downstream genes by transporting RA to combine with the retinoic acid receptor (RAR) in the nucleus [23]. Abnormal gene expression of CRABP2 has been observed in various tumors and may play an important role in regulating proliferation, apoptosis, invasion, metastasis and other activities of tumor cells [24]. The CRABP2 gene may serve as an inhibitory or promoting factor in the occurrence and development of different tumors, while its specific function and mechanism still need further study [25–30]. Considering the controversial role of CRABP2 in different tumors, our previous pan-cancer analysis supported an association between high CRABP2 expression and adverse outcomes in breast, lung, and ovarian cancers, and the high level of CRABP2 promotes docetaxel resistance in breast cancer [31].

There is currently limited research on the relationship between CRABP2 gene and immunity. The ImmPort Shared Data database (https://www.immport.org/home) includes CRABP2 as an immune related gene, and CRABP2 has been identified as a tumor mutation burden (TMB)-related immune gene for melanoma [32,33]. In melanoma, the expression level of CRABP2 in the high-TMB group was significantly higher than that in the low-TMB group, and was associated with poor prognosis. And the copy number variation (CNVs) of CRABP2 gene can inhibit the immune infiltration level of B cell, T Cell, macrophage, neutrophil and dendritic cell [33]. Since the role of CRABP2 gene in tumor immunotherapy has not been elucidated, we conducted a correlation analysis between the expression of CRABP2 and the efficacy of immunotherapy. Through bioinformatics analysis, we found that the level of CRABP2 may be related to the response of melanoma to PD-1 and may become a new target to improve the efficacy of immunotherapy by influencing immune infiltration of CAFs.

## Materials and Methods

### Expression of CRABP2 in tumor cells and tissues

The differences of CRABP2 expression between normal and tumor cell lines were compared in the GENT2 platform (http://gent2.appex.kr/gent2/), basing on gene expression profiles across cancer experiments from GPL570 platform [34]. In addition, the GEPIA2.0 website was utilized to compare the differences in CRABP2 expression between tumor and normal tissue in the TCGA and GTEx databases, and the cutoff value of |log2 (Fold Change)| defaults to 1 (http://gepia2.cancer-pku.cn/#analysis) [35].

### Correlation between the expression levels of CRABP2 and checkpoint molecules

A comprehensive correlation analysis between expression of CRABP2 and expression of checkpoint molecules, including PD-1 (PDCD1 gene), PD-L1 (CD274 gene) and CTLA-4 (CTLA4 gene), in tumors of TCGA database were executed by the “Gene_Corr” module of the TIMER2.0 website (http://timer.comp-genomics.org/), and the results were presented by a heat map [36]. Similarly, the correlation between CRABP2 and checkpoint molecules in various tumors from TCGA was also identified using Spearman methods in the Xiantao tool (https://www.xiantao.love/) [37].

### Effect of CRABP2 level on prognosis and response of ICIs in tumor

The “Immunotherapy” module of the Kaplan-Meier Plotter (https://kmplot.com/analysis/) [38] was used to analyze the effects of CRABP2 expression on overall survival (OS) and progression-free survival (PFS) of tumor patients treated with ICIs. The tumor types included bladder carcinoma, esophageal adenocarcinoma, glioblastoma, hepatocellular carcinoma, head and neck squamous cell carcinoma, melanoma, lung cancer and urothelial tumor. In addition, the Receiver Operating Characteristic (ROC) test in the “Immunotherapy of clinical samples” module of the ROC plotter website (https://www.rocplot.org/cells) [39] was employed to formalize the association between CRABP2 levels and the response to ICIs in sixteen common tumors. All patients receiving three different ICIs were grouped into responders and non-responders basing on the calculated response in the ROC plotter website. A web portal of tumor immunotherapy gene expression resource named TIGER (http://tiger.canceromics.org/) [40] also provided information on the prognosis of patients and drug responses to ICIs in various tumors from different data sources.

### Immune infiltrates analysis

The single-cell RNA-seq data from the TISCH2 website (http://tisch.comp-genomics.org) [41] were employed to examine the CRABP2 expression in the TME of tumor treated with immunotherapy. Moreover, the ESTIMATE algorithm was used to estimate the ratio of immune components and matrix components of TME in tumor samples, with ImmunoScore, StromalScore and ESTIMATEScore as the results. These three scores were positively correlated with the proportion of immune cell, the proportion of stromal cells and the sum of the two [42]. Associations between CRABP2 expression and estimated StromalScore in TCGA dataset and multiple GEO datasets were obtained from the “Cell infiltration” module of the BEST website (https://rookieutopia.com/).

The EPIC, MCPCOUNTER and TIDE algorithms provided by the “Immune_Gene” module of the TIMER2 website were applied to predict the relationship with expression of CRABP2 in TCGA tumor and the infiltration of CAFs. In addition, the Spearman correlation coefficient between the expression of CRABP2 and the expression of typical phenotypic markers of CAFs were calculated using the Xiantao tool and GEPIA2 tool.

### Cell cultures and transfection

Human melanoma cell line A375 was acquired from the Cancer Research Institute of Central South University, Changsha, Hunan, China and cultured in RPMI-1640 medium (BasalMedia, Shanghai, China) comprising 1% penicillin-streptomycin (Gibco, Waltham, MA, USA) and 10% fetal bovine serum (Gibco, Waltham, MA, USA). The environment of incubator was maintained at 37°C and 5% CO_2_. Human complementary DNA (cDNA) plasmids of CRABP2 and control plasmids were obtained from Sangon Biotech Co., Ltd., Shanghai, China. The plasmids were transfected into A375 cells with Lipofectamine 3000 (Invitrogen, Carlsbad, CA, USA).

### Western blot

The specific method of western blot was consistent with our previous research [31]. Cell lysates were prepared using RIPA lysis buffer (Bimake, Houston, Texas, USA), and the proteins in cell lysates were separated using 10% SDS–PAGE and transferred into PVDF membranes (Millipore, Billerica, MA, USA). After being blocked with skimmed dry milk, PVDF membranes were incubated overnight with primary antibodies of CRABP2 (Proteintech, Chicago, Illinois, USA) and β-Actin (Santa Cruz, Dallas, Texas, USA). Later, Immobilon Western chemiluminescent reagents (Millipore, billerica, MA, USA) were used to detect proteins on the membranes.

### Animal experimentation

The animal experiments in this study were approved by the Animal Experimental Ethics Committee of Central South University, Changsha, Hunan, China (Approval number: CSU-2023-0202). The 4-week-old female BALB/C-nu/nu mice were randomly divided into 2 groups with 5 mice per group. Mice were injected subcutaneously with A375 cells (2 × 10^5^) and their body weight was recorded daily. After 7 days from cell injection, the tumor volume was monitored every day with vernier calipers, calculating as V = (L × W^2^)/2 (V, volume; L, length; W, width). After 7 days of observation, the mice were euthanized and subcutaneous tumor tissues were collected.

### Functional enrichment analysis

The gene set enrichment analysis (GSEA) of CRABP2-related genes was carried out using the Xiantao tool to determine the potential signaling pathway of CRABP2 participated in the regulation of melanoma. Furthermore, for melanoma treated with immunotherapy, the TISCH2 website also enriched signal pathway of up-regulated and down-regulated gene-sets derived from the Kyoto Encyclopedia of Genes and Genomes Pathway-based Enrichment Analysis (KEGG).

### Statistical analysis

SPSS 23.0 was applied for data analysis and GraphPad Prism 8 was conducted for data charting. Student’s *t*-test was performed to compare the mean equality of two independent samples. For all results, *p < 0.05; **p < 0.01; ***p < 0.001.

## Results

### Associations of CRABP2 expression and checkpoint molecules in tumors

**Figure 1:**
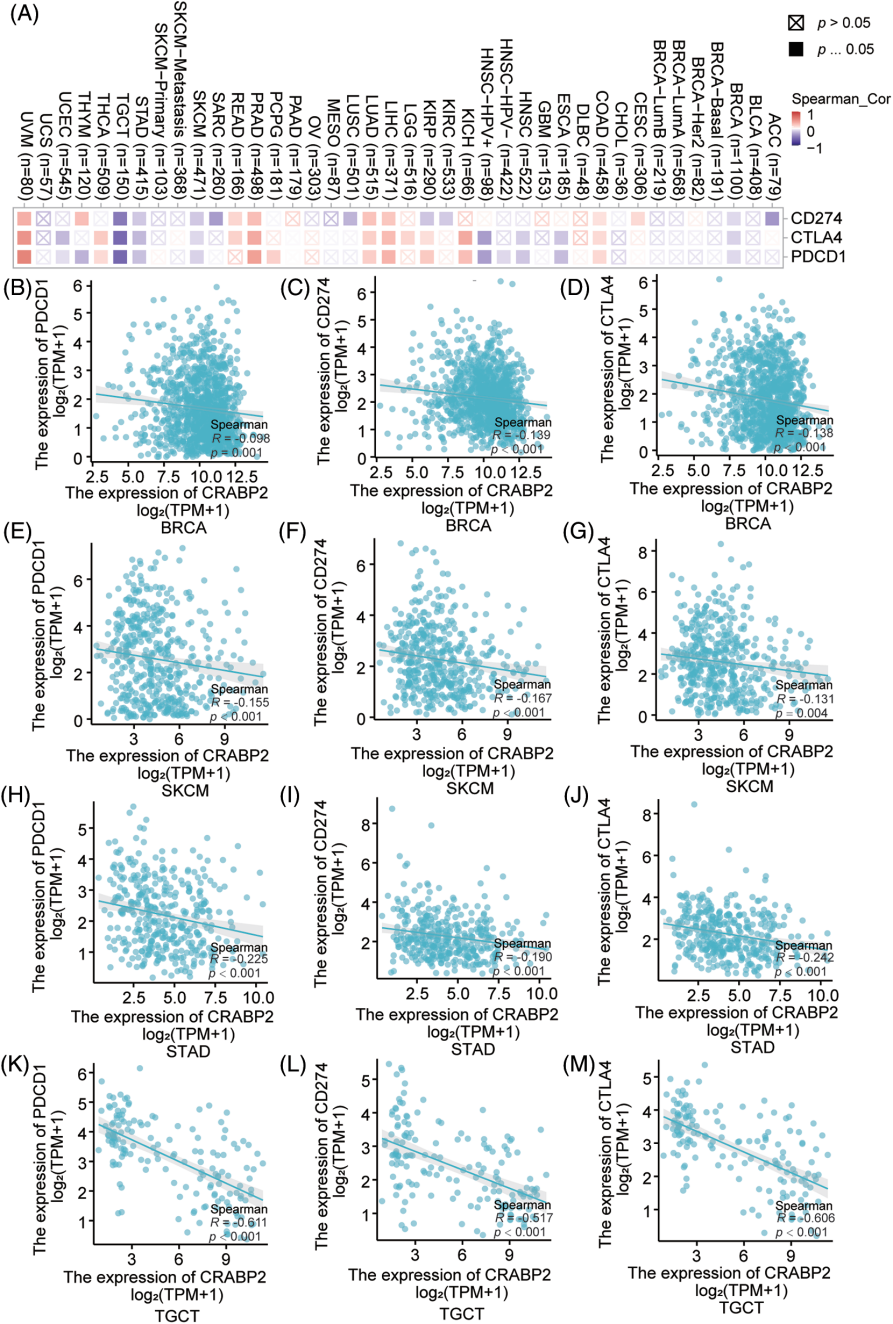
Associations of CRABP2 expression with checkpoint molecules in human tumors. (A) Associations of mRNA expression between CRABP2 and three immune checkpoint molecules (PDCD1, CD274 and CTLA4) in TCGA tumor that analyzed by TIMER2.0. (B–M) Associations of mRNA expression between CRABP2 and three immune checkpoint molecules (PDCD1, CD274 and CTLA4) in breast invasive carcinoma (BRCA) (B–D), skin cutaneous melanoma (SKCM) (E–G), stomach adenocarcinoma (STAD) (H–J) and testicular germ cell tumors (TGCT) (K–M) that analyzed by Xiantao.

The associations of mRNA expression between CRABP2 and three immune checkpoint molecules (PDCD1, CD274 and CTLA4) were examined in 33 different tumor types from the TCGA database using the TIMER2.0 and Xiantao tools. As shown in Fig. 1A, the correlation between CRABP2 and checkpoint molecules in all tumor types were displayed in a heat map. Further validation through the TIMER2.0 and Xiantao tool indicated that there were significant negative correlations in both online tools between CRABP2 and three checkpoint molecules in breast invasive carcinoma (BRCA), skin cutaneous melanoma (SKCM), stomach adenocarcinoma (STAD) and testicular germ cell tumors (TGCT) (Figs. 1B–1M). On the contrary, both tools suggested that the above molecules were significantly positively correlated in liver hepatocellular carcinoma (LIHC), lung adenocarcinoma (LUAD), prostate adenocarcinoma (PRAD) and uveal melanoma (UVM) (Suppl. Fig. S1).

### Prognostic value and efficacy prediction of CRABP2 in immunotherapy treatment

**Figure 2:**
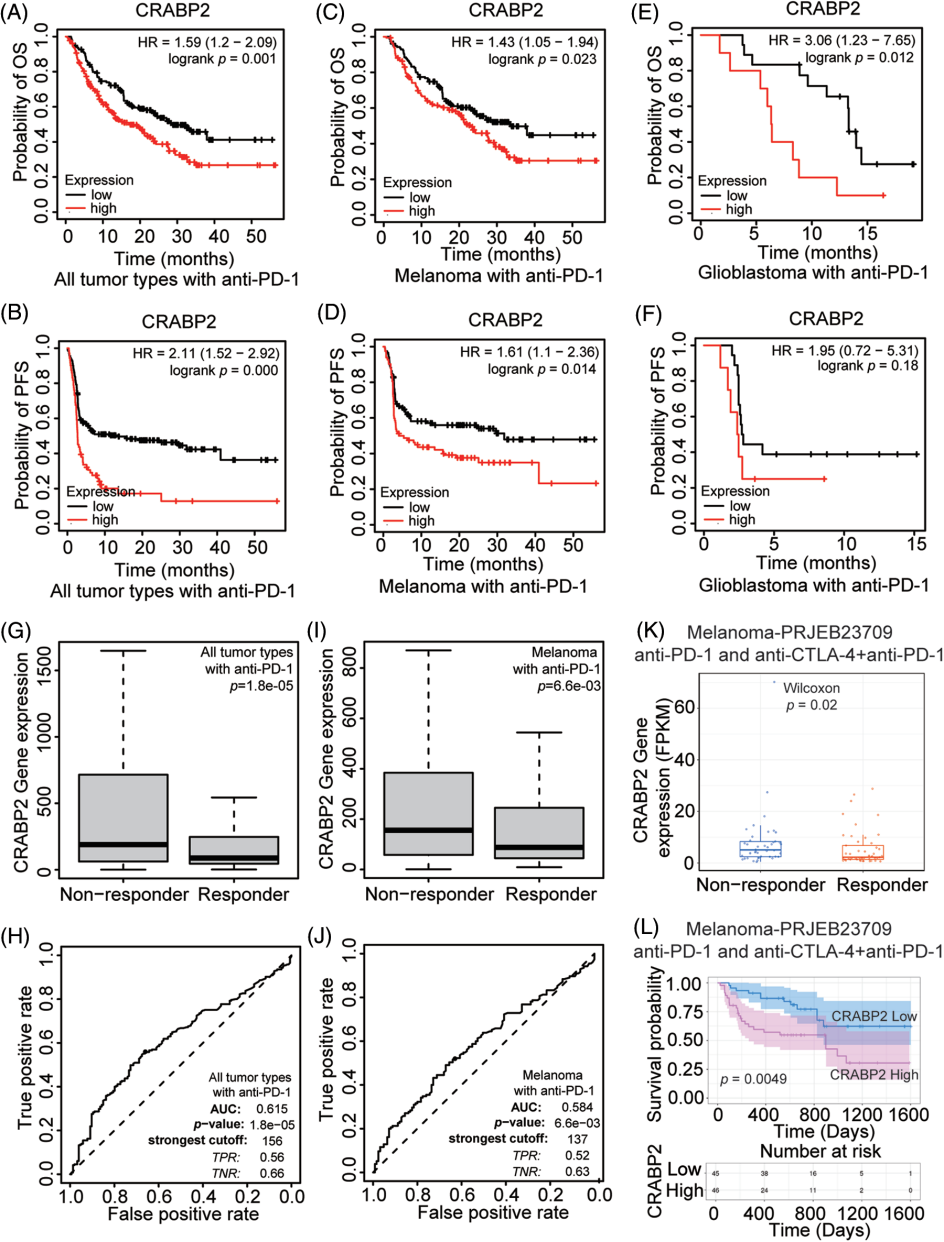
Prognostic value and efficacy prediction of CRABP2 in immunotherapy. Relationship between the expression level of CRABP2 and prognosis in all tumor patients (A and B), melanoma patients (C and D) and glioblastoma patients (E and F) receiving PD-1 inhibitors which analyzed by Kaplan-Meier plotter. Expression level of CRABP2 in PD-1 non-responder group and PD-1 responder group in both all tumor patients (G) and melanoma patients (I) that analyzed by ROC plotter. AUC value of sensitivity prediction for PD-1 inhibitors basing on CRABP2 for all tumor patients (H) and melanoma patients (J). (K) Expression level of CRABP2 in non-responder group and responder group of melanoma patients accepted immunotherapy that analyzed by TIGER. (L) Relationship between the expression level of CRABP2 and prognosis in melanoma patients receiving immunotherapy which analyzed by TIGER.

The Kaplan-Meier plotter website was used to analyze the relationship between the expression level of CRABP2 and prognosis in tumor patients receiving ICIs. It was found that for all patients and melanoma patients receiving PD-1 inhibitors included in the website, higher level of CRABP2 predicted adverse OS and PFS (Figs. 2A–2D). Moreover, an association between higher expression of CRABP2 and poor OS was observed in glioblastoma, but there was no significant difference in PFS (Figs. 2E and 2F). For patients receiving PD-L1 inhibitors, a significant association was observed between low level of CRABP2 and poor PFS in all patients and esophageal adenocarcinoma patients, but no association with OS was found. No significant correlation between CRABP2 and prognosis was observed in patients receiving CTLA-4 inhibitors, regardless of overall or specific tumor types (Suppl. Fig. S2).

To explore the potential predictive significance of CRABP2 in response to immunotherapy, the ROC plotter was used to analyze the correlation between the expression of CRABP2 and the sensitivity of three ICIs in tumors. The results showed that in all tumor types and melanoma patients accepted anti-PD-1 therapy, the expression of CRABP2 was significantly increased in nonresponsive group (all tumor type: *p* = 0.000, Fig. 2G, ROC curve in Fig. 2H; melanoma: *p* = 0.007, Fig. 2I, ROC curve in Fig. 2J). The ROC curves showed that the values of area under the curve (AUC) predicted by CRABP2 for the sensitivity of PD-1 inhibitors in all tumor types and melanoma were 0.615 and 0.584. No significant differences of sensitivity were found in tumor patients receiving PD-L1 inhibitors and CTLA4 inhibitors. In addition, in a transcriptome dataset from the TIGER website that included melanoma patients receiving PD-1 inhibitors or receiving PD-1 inhibitors combined with CTLA4 inhibitors [43], expression of CRABP2 was significantly higher in the non-responder group, and high level of CRABP2 gene suggested poor survival probability (Figs. 2K and 2L). Based on the above results of CRABP2 on the prediction of efficacy and prognosis in immunotherapy, we found that in melanoma, the high expression of CRABP2 may be related to the downregulation of checkpoint molecules, contributing to non-response and poor-prognosis to PD-1 treatment.

### The influence of CRABP2 on immune infiltration in SKCM

**Figure 3:**
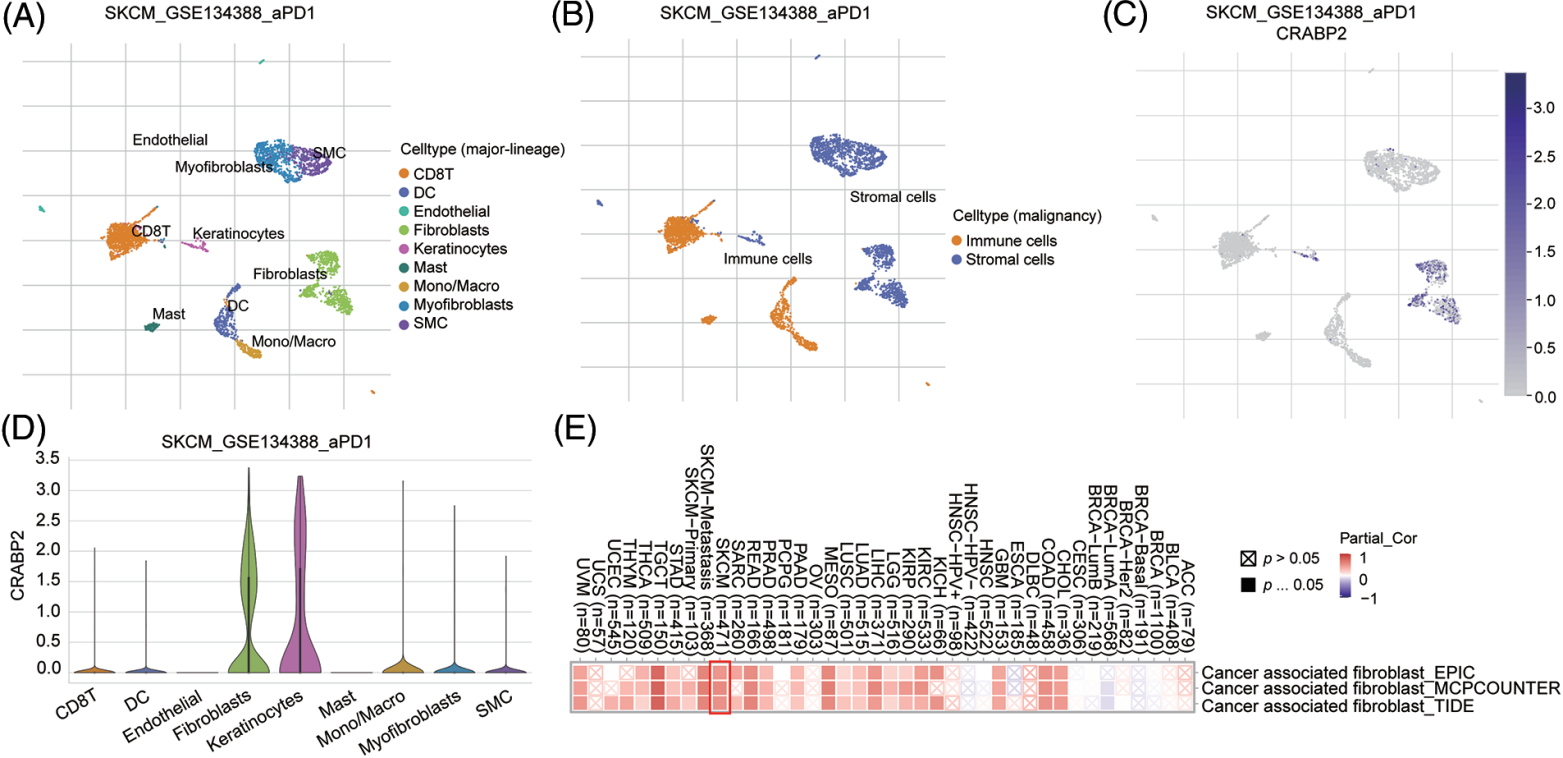
The influence of CRABP2 on immune infiltration in SKCM. (A and B) The immune cells, stromal cells, and specific cell types at the single-cell level of advanced melanoma patients with good response to PD-1 inhibitors which were analyzed on TISCH2. Umap (C) and violin plot (D) to explore the immune characteristics of CRABP2 gene in melanoma which responded well to PD-1 inhibitors which were analyzed on TISCH2. (E) Correlation analysis between CRABP2 and cancer-associated fibroblasts (CAFs) across different cancers in TCGA.

We attempted to explore the mechanism by which CRABP2 affects the efficacy of PD-1 inhibitors in melanoma at the single-cell level. Single cell RNA-seq data of advanced melanoma patients with good response to PD-1 inhibitors in GSE134388 dataset were analyzed on TISCH2 website [44]. Umap format was used to display immune cells, stromal cells, and specific cell types including CD8 T cell, DC cell, endothelial cell, fibroblasts cell, etc. (Figs. 3A and 3B). Umap and violin plot were used to explore the immune characteristics of CRABP2 gene in melanoma which responded well to PD-1 inhibitors. It was found that the expression of CRABP2 in fibroblasts was significantly increased (Figs. 3C and 3D). Subsequently, three different algorithms on the TIMER2 website were used to demonstrate the effect of CRABP2 on CAFs infiltration in TCGA tumor, indicating a positive correlation between CRABP2 and CAFs infiltration in SKCM (spearman’s R: 0.481–0.545, *p* < 0.05) (Fig. 3E).

**Figure 4:**
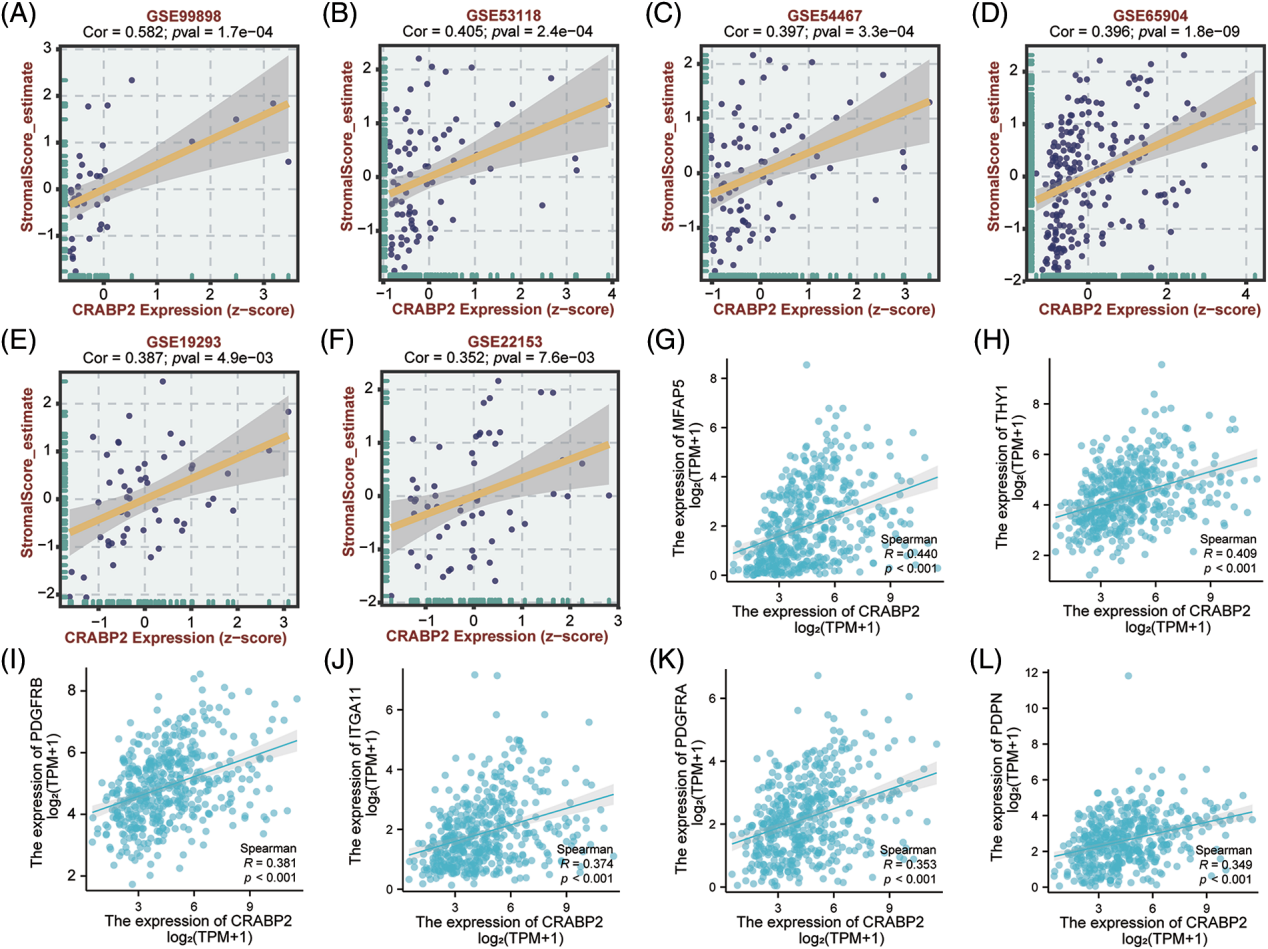
Relationship between CRABP2 expression level and infiltration of CAFs in SKCM. Correlation between CRABP2 expression and estimated StromalScore in SKCM from GES99898 (A), GSE53118 (B), GSE54467 (C), GSE65904 (D), GSE19293 (E) and GSE22153 (F) which analyzed by BEST. (G–L) Correlation analyses between CRABP2 and the CAFs markers including MFAP5, THY1, PDGFRα/β, ITGA11 and PDPN by Xiantao.

Significant positive correlations between CRABP2 expression and estimated StromalScore were observed in SKCM from TCGA data sets and nine GEO data sets. The correlation coefficients were greater than 0.3 in six GEO data sets (Figs. 4A–4F), while the correlation coefficients of other data sets were greater than 0.2 (Suppl. Fig. S3). Then, two tools, Xiantao and GEPIA2.0, were used to analyze the correlation between CRABP2 and typical phenotypic markers of CAFs in SKCM samples of TCGA, (such as FAP, ACTA2, MFAP5, COL11A1, Tenascin-C, PDPN, ITGA11, NG2, CXCL12, S100A4, PDGFRα/β, POSTN, CD74, THY1, Vimentin and other markers) [45–48]. Among them, both calculations showed that FAP, ACTA2, MFAP5, COL11A1, Tenascin-C, PDPN, ITGA11, S100A4, PDGFRα/β, POSTN, THY1 molecules were significantly positively correlated with CRABP2 in SKCM. Especially for MFAP5, PDPN, ITGA11, PDGFRα/β, THY1, the correlation coefficients were all greater than 0.3 (Figs. 4G–4L). The above results suggested that CRABP2 may be related to immune infiltration of CAFs in patients with SKCM.

### CRABP2 accelerated tumor growth in SKCM in vivo

**Figure 5:**
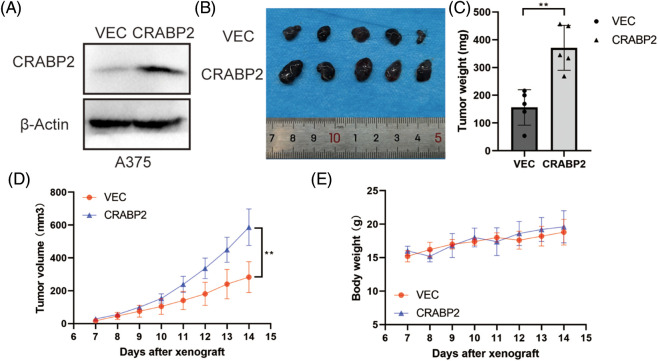
CRABP2 accelerated tumor growth of SKCM in BALB/C-nu/nu mice. (A) After transfection with control plasmids or cDNA plasmids of CRABP2 in A375 cells, the protein level of CRABP2 were detected by western blot. (B) Tumors isolated from the skin of mice were photographed. (C) The tumors of each category were weighed and compared, respectively. (D) The tumor volumes were calculated according to the time interval of tumor cell injection, and the calculation formula was V = (L × W^2^)/2 (V, volume; L, length; W, width). (E) The weights of mice were recorded according to the time interval of tumor cell injection. * *p* < 0.05; **p < 0.01.

By investigating the CRABP2 levels in cell lines and tissues of melanoma using GENT2 and GEPIA2 tool, we found that the CRABP2 expression in normal samples was significantly higher than that in tumor-cell lines and tissues (Suppl. Fig. S4). Therefore, to verify the relationship of CRABP2 and the prognosis of melanoma patients, we tested the tumorigenicity of melanoma cells with overexpression of CRABP2 *in vivo*. Following overexpression of cDNA plasmids of CRABP2 in A375 melanoma cells (Fig. 5A), melanoma cells were injected into BALB/C-nu/nu mice to create subcutaneous melanoma models. As shown in Figs. 5B–5E, compared to the control group, the mice models derived from melanoma cells with CRABP2 overexpression had significantly increased tumor weight and volume, while the body weight of mice did not have a significant difference.

### Role of CRABP2 in CAFs from SKCM

**Figure 6:**
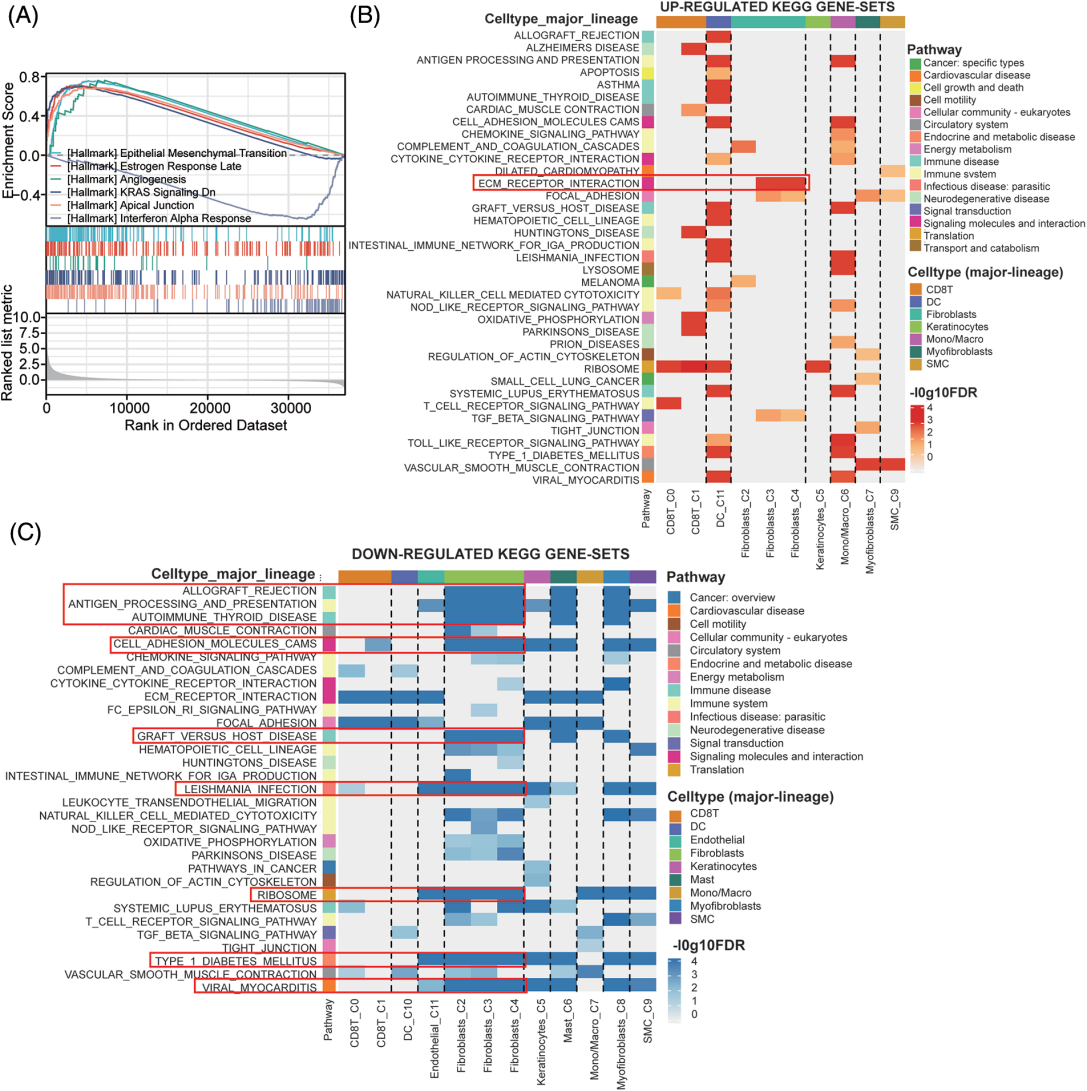
The effects of CRABP2 in regulating CAFs in SKCM. (A) GSEA analysis of CRABP2-related genes for SKCM in TCGA which conducted in Xiantao. (B and C) KEGG pathway analysis in SKCM patients who responded well to PD-1 inhibitors provided by TISCH2.

To explore the potential signaling pathway of CRABP2 involved in immune regulation of SKCM, relevant genes obtained from the differential analysis of CRABP2 gene were used to conduct GSEA. The epithelial-mesenchymal transition (EMT) pathway had the highest enrichment score in GSEA, suggesting that CRABP2 tend to participate in the control of EMT in SKCM (Fig. 6A). We previously observed high expression of CRABP2 in fibroblasts during single-cell analysis of SKCM, and then conducted KEGG pathway analysis using single-cell data provided by the TISCH2 website. The results showed that significant upregulation of ECM-receptor interaction was observed in fibroblasts from SKCM patients who responded well to PD-1 inhibitors (Fig. 6B). On the contrary, multiple pathways, including allograft rejection, antigen processing and presentation, autoimmune thyroid disease, cell adhesion molecules (CAMs), graft *vs*. host disease, leishmania infection, ribosome, type1 diabetes mellitus, and viral myocarditis, were downregulated in fibroblasts (Fig. 6C).

## Discussion

Considering the limited research on CRABP2 and tumor immunity, we attempted to apply bioinformatics analysis to explore the potential significance of CRABP2 for tumor immunotherapy. After repeated validation in different tools, we noted that the expression of CRABP2 in BRCA, SKCM, STAD and TGCT was significantly negatively correlated with the level of immune checkpoint molecules. Our previous studies have found that the expression of CRABP2 in BRCA was significantly higher than that in adjacent normal tissues, indicating an association to poor clinical outcome and docetaxel resistance [31]. CRABP2 was identified as a TMB-related gene in SKCM, and the significance of CRABP2 for tumor prognosis was attempted to analyze from two immune-related aspects: TMB and immune infiltration [33]. In STAD, CRABP2 was highly expressed under TET1-mediated DNA hydroxymethylation, which was correlated with poor OS and promoted oxaliplatin resistance [48]. There is currently no research focusing on the role of CRABP2 in TGCT. Based on our findings and previous research reports, we have found that CRABP2 may be associated with the prognosis of multiple tumors, particularly affecting the immunotherapeutic efficacy and immune infiltration of SKCM.

Under the circumstances, we found that patients with higher levels of CRABP2 in SKCM treated with PD-1 inhibitors predicted poor prognosis, and the expression of CRABP2 was significantly elevated in SKCM patients who did not respond to PD-1 inhibitor treatment. The above results indicated the potential of CRABP2 in predicting the efficacy of PD-1 inhibitors in SKCM. All trans retinoic acid (ATRA) had the ability to activate innate and adaptive immunity, and signaling pathways mediated by retinoic acid and RAR were involved in immune-modulatory response of skin [49]. Our findings were like previous research reports in which the expression level of CRABP2 was significantly increased in the high-TMB group compared to the low-TMB group, and high level of CRABP2 was associated with adverse survival outcomes in SKCM patients [33]. In addition, CRABP2 was also differentially expressed as an immune related gene in clear cell renal cell carcinoma and was associated with poor prognosis. In the risk score model constructed with participation of CRABP2, it was found that the unfavorable-risk group had higher immune infiltration (CD8 T cell, Treg cell and NK cell) and higher expression of immune response suppressors (PD-1, CTLA4, TNFRSF9, TIGIT and LAG3) [50].

For SKCM, we also found a significant positive correlation between the expression of CRABP2 and StromalScore, which represents stromal cells, in different data sets of TCGA and GEO. Single-cell analysis of SKCM patients responding to PD-1 inhibitors suggested a significant increase of CRABP2 level in fibroblasts. The immune infiltration analysis of stromal cells in TME also showed a positive association between CRABP2 and CAFs infiltration in SKCM. Moreover, it was found that CRABP2 was significantly positively correlated with typical phenotypic markers of CAFs in SKCM, such as MFAP5, PDPN, ITGA11, PDGFRα/β and THY1. The results of animal experiments had also demonstrated that CRABP2 overexpression promoted tumorigenesis of melanoma, and the underlying mechanism was worthy of further verification in mouse models. It was reported that through immunohistochemical staining and immunofluorescence assay, it was found that fibroblasts in skin samples expressed CRABP2 [51]. Previous studies have observed a correlation between the expression of CAFs and CRABP2 in pancreatic ductal adenocarcinoma (PDAC), where the two transporters of ATRA, fatty acid binding protein 5 (FABP5) and CRABP2, always appear in a high FABP5: CRABP2 ratio in quiescent fibroblasts. When the pancreatic stellate cells (PSC) transformed into CAFs (activated fibroblasts) after RA deficiency, Hughes et al. observed that the level of CRABP2 and FABP5 in both PSC and CAFs were significantly lower than that in quiescent fibroblasts [30].

The results of pathway enrichment suggested that the potential signaling pathways of CRABP2 involved in immune regulation of SKCM were concentrated in EMT and ECM-receptor interaction. CRABP2 has been reported to promote EMT of tumor cells in different tumors. In thyroid cancer cells, the expression of CRABP2 was upregulated by LINC01816, thus promoting EMT of tumor [52]. In non-small cell lung cancer cells, inhibition of CRABP2 may inhibited the NF-κB pathway, leading to a decrease in EMT of tumor cells [53]. In ER negative breast cancer cells, CRABP2 induced downregulation of Hippo pathway to promote EMT, but in ER positive breast cancer cells, CRABP2 had opposite effect on EMT [26]. Moreover, CRABP2 had been observed in serous ovarian cancer cells to enhance TRIM16 methylation through upregulation of EZH2, resulting in enhanced EMT effect [54]. The current mainstream idea is that CAFs promoted tumor invasion by releasing transforming growth factor-β (TGFβ) to activate EMT in tumor cells [55,56]. It is also observed in breast cancer cells that IL-32 secreted by CAFs activated p38 MAPK signaling to promote EMT [57]. At present, no correlation between CRABP2 and ECM had been reported. The main role of CAFs in the changes of ECM was to promote the synthesis and release of a large number of ECM proteins, and affect the remodeling of ECM [58,59]. Therefore, combined with our research findings, we proposed that CRABP2 may affect immune infiltration of CAFs in SKCM by regulating EMT and ECM, thereby affecting the efficacy of immunotherapy in SKCM.

This study unveils a novel perspective on the involvement and importance of CRABP2 in tumor immunotherapy, particularly highlighting its role in controlling the infiltration of CAFs and the immune response in SKCM. Our profound examination for impact of CRABP2 on immunotherapy revolutionizes its potential as a biomarker for immunotherapy. However, the study was somewhat constrained by its reliance on bioinformatics analysis, thereby limiting the confirmation of precise regulatory pathways for CRABP2 in the immunotherapy of SKCM. Subsequent investigations will further delve into this aspect to enhance our understanding.

## Conclusion

This study unveiled a negative association between expression of CRABP2 and expressions of immune checkpoint markers in SKCM. Especially in SKCM patients treated with PD-1 inhibitors, a notable increase in CRABP2 expression was observed and associated with poor prognostic outcomes. Responders to PD-1 inhibitors displayed heightened levels of CRABP2 in their fibroblasts. Additionally, CRABP2 was found to promote tumor growth in SKCM xenograft mice and positively correlate with infiltration and marker of CAFs in SKCM. CRABP2 may also influence immune regulation in SKCM through its influence on EMT and ECM. These finding contributes to understanding the potential of CRABP2 as a biomarker for PD-1 inhibitors. This study suggests that CRABP2 has the potential to impact the sensitivity to PD-1 inhibitors by modulating CAFs infiltration, offering a new target for improving the efficacy and responsiveness of PD-1 inhibitors in SKCM patients. The methods utilized in this study chiefly relied on bioinformatics analysis, thus future research should complement these findings with additional experimental data to elucidate the specific regulatory pathways governing the immune efficacy and CAFs infiltration mediated by CRABP2 in SKCM.

## Supplementary Materials

Supplemental Figure S1 Associations of CRABP2 expression with checkpoint molecules in human tumors. (A-L) mRNA expression associations between CRABP2 and three immune checkpoint molecules (PDCD1, CD274 and CTLA4) in LIHC (A-C), LUAD (D-F), PRAD (G-I), UVM (J-L) tumor that analyzed by Xiantao.

Figure S2 Prognostic value of CRABP2 in immunotherapy. Relationship between the expression level of CRABP2 and prognosis in all tumor patients (A-B) and esophageal adenocarcinoma patients (C-D) receiving PD-L1 which analyzed by Kaplan-Meier plotter. Relationship between the expression level of CRABP2 and prognosis in all tumor patients (E-F) and melanoma patients (G-H) receiving CTLA-4 which analyzed by Kaplan-Meier plotter.

Figure S3 Correlation between CRABP2 expression and estimated StromalScore in SKCM data sets which analyzed by BEST. (A) GES190113, (B) GSE59455, (C) GSE46517 and (D) TCGA.

Figure S4 Expression of CRABP2 in tumor cells and tissues. (A) Gene expression of CRABP2 in normal and tumor cell lines that analyzed by GENT2. (B) mRNA expression level of CRABP2 in normal and tumor tissues of SKCM from TCGA and GTEx datasets that analyzed by GEPIA2. Red asterisks indicate significant differences.

## Data Availability

Data is available upon reasonable request.

## Supplementary Materials

The supplementary material is available online at https://doi.org/10.32604/or.2023.042345.

## References

[1] Bai, R., Yuan, C. (2022). Kita-kyushu lung cancer antigen-1 (KK-LC-1): A promising cancer testis antigen. Aging and Disease*,* 13*,* 1267–1277; 35855340 10.14336/AD.2021.1207 PMC9286905

[2] Yang, L., Liu, G., Li, Y., Pan, Y. (2022). The emergence of tumor-infiltrating lymphocytes in nasopharyngeal carcinoma: Predictive value and immunotherapy implications. Genes & Diseases*,* 9*,* 1208–1219. 35873027 10.1016/j.gendis.2021.07.002 PMC9293699

[3] Alausa, A., Victor, U. C., Fadahunsi, O. S., Owolabi, N., Adeniji, A. et al. (2022). Checkpoints and immunity in cancers: Role of GNG12. Pharmacological Research*,* 180*,* 106242; 35513227 10.1016/j.phrs.2022.106242

[4] Hagemans, I. M., Wierstra, P. J., Steuten, K., Molkenboer-Kuenen, J. D. M., van Dalen, D. et al. (2022). Multiscale imaging of therapeutic anti-PD-L1 antibody localization using molecularly defined imaging agents. Journal of Nanobiotechnology*,* 20*,* 64; 35109860 10.1186/s12951-022-01272-5 PMC8811974

[5] Ou, L., Wang, H., Huang, H., Zhou, Z., Lin, Q. et al. (2022). Preclinical platforms to study therapeutic efficacy of human gammadelta T cells. Clinical and Translational Medicine*,* 12*,* e814; 35731974 10.1002/ctm2.814 PMC9217106

[6] Zhang, H., Dai, Z., Wu, W., Wang, Z., Zhang, N. et al. (2021). Regulatory mechanisms of immune checkpoints PD-L1 and CTLA-4 in cancer. Journal of Experimental & Clinical Cancer Research*,* 40*,* 184. 34088360 10.1186/s13046-021-01987-7 PMC8178863

[7] Rotte, A. (2019). Combination of CTLA-4 and PD-1 blockers for treatment of cancer. Journal of Experimental & Clinical Cancer Research*,* 38*,* 255. 31196207 10.1186/s13046-019-1259-z PMC6567914

[8] Postow, M. A., Callahan, M. K., Wolchok, J. D. (2015). Immune checkpoint blockade in cancer therapy. Journal of Clinical Oncology*,* 33*,* 1974–1982; 25605845 10.1200/JCO.2014.59.4358 PMC4980573

[9] Shklovskaya, E., Rizos, H. (2020). Spatial and temporal changes in PD-L1 expression in cancer: The role of genetic drivers, tumor microenvironment and resistance to therapy. International Journal of Molecular Sciences*,* 21*,* 7139; 32992658 10.3390/ijms21197139 PMC7583014

[10] Wang, S., Jiang, M., Yang, Z., Huang, X., Li, N. (2022). The role of distinct co-mutation patterns with TP53 mutation in immunotherapy for NSCLC. Genes & Diseases*,* 9*,* 245–251. 35005121 10.1016/j.gendis.2020.04.001 PMC8720680

[11] Huang, Y., Lai, H., Jiang, J., Xu, X., Zeng, Z. et al. (2022). pH-activatable oxidative stress amplifying dissolving microneedles for combined chemo-photodynamic therapy of melanoma. Asian Journal of Pharmaceutical Sciences*,* 17*,* 679–696; 36382300 10.1016/j.ajps.2022.08.003 PMC9640714

[12] Villani, A., Potestio, L., Fabbrocini, G., Troncone, G., Malapelle, U. et al. (2022). The treatment of advanced melanoma: Therapeutic update. International Journal of Molecular Sciences*,* 23*,* 6388; 35742834 10.3390/ijms23126388 PMC9223461

[13] Chu, Y., Qian, L., Ke, Y., Feng, X., Chen, X. et al. (2022). Lymph node-targeted neoantigen nanovaccines potentiate anti-tumor immune responses of post-surgical melanoma. Journal of Nanobiotechnology*,* 20*,* 190; 35418151 10.1186/s12951-022-01397-7 PMC9006542

[14] Sui, Y., Li, J., Qu, J., Fang, T., Zhang, H. et al. (2022). Dual-responsive nanovaccine for cytosolic delivery of antigens to boost cellular immune responses and cancer immunotherapy. Asian Journal of Pharmaceutical Sciences*,* 17*,* 583–595; 36101894 10.1016/j.ajps.2022.05.004 PMC9459061

[15] Lisi, L., Lacal, P. M., Martire, M., Navarra, P., Graziani, G. (2022). Clinical experience with CTLA-4 blockade for cancer immunotherapy: From the monospecific monoclonal antibody ipilimumab to probodies and bispecific molecules targeting the tumor microenvironment. Pharmacological Research*,* 175*,* 105997; 34826600 10.1016/j.phrs.2021.105997

[16] Xing, S., Hu, K., Wang, Y. (2022). Tumor immune microenvironment and immunotherapy in non-small cell lung cancer: Update and new challenges. Aging and Disease*,* 13*,* 1615–1632; 36465180 10.14336/AD.2022.0407 PMC9662266

[17] Papait, A., Romoli, J., Stefani, F. R., Chiodelli, P., Montresor, M. C. et al. (2022). Fight the cancer, hit the CAF! Cancers*,* 14*,* 3570; 35892828 10.3390/cancers14153570 PMC9330284

[18] Wu, F., Yang, J., Liu, J., Wang, Y., Mu, J. et al. (2021). Signaling pathways in cancer-associated fibroblasts and targeted therapy for cancer. Signal Transduction and Targeted Therapy*,* 6*,* 218; 34108441 10.1038/s41392-021-00641-0 PMC8190181

[19] Sung, J. Y., Cheong, J. H. (2022). Prognosis-related gene signature is enriched in cancer-associated fibroblasts in the stem-like subtype of gastric cancer. Clinical and Translational Medicine*,* 12*,* e930; 35754321 10.1002/ctm2.930 PMC9234682

[20] Biffi, G., Tuveson, D. A. (2021). Diversity and biology of cancer-associated fibroblasts. Physiological Reviews*,* 101*,* 147–176; 32466724 10.1152/physrev.00048.2019 PMC7864232

[21] Mao, X., Xu, J., Wang, W., Liang, C., Hua, J. et al. (2021). Crosstalk between cancer-associated fibroblasts and immune cells in the tumor microenvironment: New findings and future perspectives. Molecular Cancer*,* 20*,* 131; 34635121 10.1186/s12943-021-01428-1 PMC8504100

[22] Galbo Jr, P. M., Zang, X., Zheng, D. (2021). Molecular features of cancer-associated fibroblast subtypes and their implication on cancer pathogenesis, prognosis, and immunotherapy resistance. Clinical Cancer Research*,* 27 *(*9*),* 2636–2647; 33622705 10.1158/1078-0432.CCR-20-4226 PMC8102353

[23] Lixa, C., Clarkson, M. W., Iqbal, A., Moon, T. M., Almeida, F. C. L. et al. (2019). Retinoic acid binding leads to CRABP2 rigidification and dimerization. Biochemistry*,* 58*,* 4183–4194; 31566355 10.1021/acs.biochem.9b00672 PMC13047426

[24] Jiao, X., Liu, R., Huang, J., Lu, L., Li, Z. et al. (2020). Cellular retinoic-acid binding protein 2 in solid tumor. Current Protein & Peptide Science*,* 21*,* 507–516. 32013828 10.2174/1389203721666200203150721

[25] Li, N., Lin, G., Zhang, Y., Zhang, Q., Zhang, H. (2022). Exosome-related protein CRABP2 is upregulated in ovarian carcinoma and enhances cell proliferation. Discover Oncology*,* 13*,* 33; 35578123 10.1007/s12672-022-00492-3 PMC9110584

[26] Feng, X., Zhang, M., Wang, B., Zhou, C., Mu, Y. et al. (2019). CRABP2 regulates invasion and metastasis of breast cancer through hippo pathway dependent on ER status. Journal of Experimental & Clinical Cancer Research*,* 38*,* 361. 31419991 10.1186/s13046-019-1345-2 PMC6697986

[27] Zhang, Y., Wang, H., Wang, J., Bao, L., Wang, L. et al. (2015). Global analysis of chromosome 1 genes among patients with lung adenocarcinoma, squamous carcinoma, large-cell carcinoma, small-cell carcinoma, or non-cancer. Cancer Metastasis Reviews*,* 34*,* 249–264; 25937073 10.1007/s10555-015-9558-0

[28] Percicote, A. P., Mardegan, G. L., Gugelmim, E. S., Ioshii, S. O., Kuczynski, A. P. et al. (2018). Tissue expression of retinoic acid receptor alpha and CRABP2 in metastatic nephroblastomas. Diagnostic Pathology*,* 13*,* 9; 29378601 10.1186/s13000-018-0686-z PMC6389245

[29] Yang, Q., Wang, R., Xiao, W., Sun, F., Yuan, H. et al. (2016). Cellular retinoic acid binding protein 2 is strikingly downregulated in human esophageal squamous cell carcinoma and functions as a tumor suppressor. PLoS One*,* 11*,* e0148381; 26839961 10.1371/journal.pone.0148381 PMC4739712

[30] Hughes, C. S., ChinAleong, J. A., Kocher, H. M. (2020). CRABP2 and FABP5 expression levels in diseased and normal pancreas. Annals of Diagnostic Pathology*,* 47*,* 151557; 32593808 10.1016/j.anndiagpath.2020.151557

[31] Zeng, S., Xu, Z., Liang, Q., Thakur, A., Liu, Y. et al. (2023). The prognostic gene CRABP2 affects drug sensitivity by regulating docetaxel-induced apoptosis in breast invasive carcinoma: A pan-cancer analysis. Chemico-Biological Interactions*,* 373*,* 110372; 36736488 10.1016/j.cbi.2023.110372

[32] Bhattacharya, S., Dunn, P., Thomas, C. G., Smith, B., Schaefer, H. et al. (2018). ImmPort, toward repurposing of open access immunological assay data for translational and clinical research. Scientific Data*,* 5*,* 180015; 29485622 10.1038/sdata.2018.15 PMC5827693

[33] Yan, J., Wu, X., Yu, J., Zhu, Y., Cang, S. (2020). Prognostic role of tumor mutation burden combined with immune infiltrates in skin cutaneous melanoma based on multi-omics analysis. Frontiers in Oncology*,* 10*,* 570654; 33240814 10.3389/fonc.2020.570654 PMC7683772

[34] Park, S. J., Yoon, B. H., Kim, S. K., Kim, S. Y. (2019). GENT2: An updated gene expression database for normal and tumor tissues. BMC Medical Genomics*,* 12*,* 101; 31296229 10.1186/s12920-019-0514-7 PMC6624177

[35] Li, C., Tang, Z., Zhang, W., Ye, Z., Liu, F. (2021). GEPIA2021: Integrating multiple deconvolution-based analysis into GEPIA. Nucleic Acids Research*,* 49*,* W242–W246; 34050758 10.1093/nar/gkab418 PMC8262695

[36] Li, T., Fu, J., Zeng, Z., Cohen, D., Li, J. et al. (2020). TIMER2.0 for analysis of tumor-infiltrating immune cells. Nucleic Acids Research*,* 48*,* W509–W514; 32442275 10.1093/nar/gkaa407 PMC7319575

[37] Chu, L., Yi, Q., Yan, Y., Peng, J., Li, Z. et al. (2022). A prognostic signature consisting of pyroptosis-related genes and SCAF11 for predicting immune response in breast cancer. Frontiers in Medicine*,* 9*,* 882763; 35646948 10.3389/fmed.2022.882763 PMC9133489

[38] Lanczky, A., Gyorffy, B. (2021). Web-based survival analysis tool tailored for medical research (KMplot): Development and implementation. Journal of Medical Internet Research*,* 23*,* e27633; 34309564 10.2196/27633 PMC8367126

[39] Tibor Fekete, J., Gyorffy, B. (2022). A unified platform enabling biomarker ranking and validation for 1562 drugs using transcriptomic data of 1250 cancer cell lines. Computational and Structural Biotechnology Journal*,* 20*,* 2885–2894; 35765648 10.1016/j.csbj.2022.06.007 PMC9198269

[40] Chen, Z., Luo, Z., Zhang, D., Li, H., Liu, X. et al. (2022). TIGER: A web portal of tumor immunotherapy gene expression resource. Genomics, Proteomics & Bioinformatics*,* 21 (2)*,* 337–348. https://www.sciencedirect.com/science/article/pii/S1672022922000997 10.1016/j.gpb.2022.08.004 PMC10626175 36049666

[41] Han, Y., Wang, Y., Dong, X., Sun, D., Liu, Z. et al. (2023). TISCH2: Expanded datasets and new tools for single-cell transcriptome analyses of the tumor microenvironment. Nucleic Acids Research*,* 51*,* D1425–D1431; 36321662 10.1093/nar/gkac959 PMC9825603

[42] Yue, Y., Zhang, Q., Sun, Z. (2021). CX3CR1 acts as a protective biomarker in the tumor microenvironment of colorectal cancer. Frontiers in Immunology*,* 12*,* 758040; 35140706 10.3389/fimmu.2021.758040 PMC8818863

[43] Gide, T. N., Quek, C., Menzies, A. M., Tasker, A. T., Shang, P. et al. (2019). Distinct immune cell populations define response to anti-PD-1 monotherapy and anti-PD-1/anti-CTLA-4 combined therapy. Cancer Cell*,* 35*,* 238–255.e236; 30753825 10.1016/j.ccell.2019.01.003

[44] Li, N., Kang, Y., Wang, L., Huff, S., Tang, R. et al. (2020). Alkbh5 regulates anti-PD-1 therapy response by modulating lactate and suppressive immune cell accumulation in tumor microenvironment. Proceedings of the National Academy of Sciences of the United States of America*,* 117*,* 20159–20170; 32747553 10.1073/pnas.1918986117 PMC7443867

[45] Nurmik, M., Ullmann, P., Rodriguez, F., Haan, S., Letellier, E. (2020). In search of definitions: Cancer-associated fibroblasts and their markers. International Journal of Cancer*,* 146*,* 895–905; 30734283 10.1002/ijc.32193 PMC6972582

[46] Miyai, Y., Esaki, N., Takahashi, M., Enomoto, A. (2020). Cancer-associated fibroblasts that restrain cancer progression: Hypotheses and perspectives. Cancer Science*,* 111*,* 1047–1057; 32060987 10.1111/cas.14346 PMC7156845

[47] Glabman, R. A., Choyke, P. L., Sato, N. (2022). Cancer-associated fibroblasts: Tumorigenicity and targeting for cancer therapy. Cancers*,* 14*,* 3906; 36010899 10.3390/cancers14163906 PMC9405783

[48] Tang, X., Liang, Y., Sun, G., He, Q., Hou, Z. et al. (2022). Upregulation of CRABP2 by TET1-mediated DNA hydroxymethylation attenuates mitochondrial apoptosis and promotes oxaliplatin resistance in gastric cancer. Cell Death & Disease*,* 13*,* 848. 36195596 10.1038/s41419-022-05299-2 PMC9532395

[49] Gericke, J., Ittensohn, J., Mihaly, J., Alvarez, S., Alvarez, R. et al. (2013). Regulation of retinoid-mediated signaling involved in skin homeostasis by RAR and RXR agonists/antagonists in mouse skin. PLoS One*,* 8*,* e62643; 23638129 10.1371/journal.pone.0062643 PMC3634743

[50] Liao, Z., Yao, H., Wei, J., Feng, Z., Chen, W. et al. (2021). Development and validation of the prognostic value of the immune-related genes in clear cell renal cell carcinoma. Translational Andrology and Urology*,* 10*,* 1607–1619; 33968649 10.21037/tau-20-1348 PMC8100830

[51] Fischer-Huchzermeyer, S., Dombrowski, A., Hagel, C., Mautner, V. F., Schittenhelm, J. et al. (2017). The cellular retinoic acid binding protein 2 promotes survival of malignant peripheral nerve sheath tumor cells. The American Journal of Pathology*,* 187*,* 1623–1632; 28502478 10.1016/j.ajpath.2017.02.021

[52] Zhao, H., Zhu, X., Luo, Y., Liu, S., Wu, W. et al. (2021). LINC01816 promotes the migration, invasion and epithelial‐mesenchymal transition of thyroid carcinoma cells by sponging miR‐34c‐5p and regulating CRABP2 expression levels. Oncology Reports*,* 45*,* 81; 33786631 10.3892/or.2021.8032 PMC8025121

[53] Meng, J. F., Luo, M. J., Li, H. B. (2021). Correlation between plasma cellular retinoic acid-binding protein 2 and proliferation, migration, and invasion of non-small-cell lung cancer cells. Critical Reviews in Eukaryotic Gene Expression*,* 31*,* 81–89; 34369716 10.1615/CritRevEukaryotGeneExpr.2021038129

[54] Xie, T., Tan, M., Gao, Y., Yang, H. (2022). CRABP2 accelerates epithelial mesenchymal transition in serous ovarian cancer cells by promoting TRIM16 methylation via upregulating EZH2 expression. Environmental Toxicology*,* 37*,* 1957–1967; 35442568 10.1002/tox.23542

[55] Fiori, M. E., di Franco, S., Villanova, L., Bianca, P., Stassi, G. et al. (2019). Cancer-associated fibroblasts as abettors of tumor progression at the crossroads of EMT and therapy resistance. Molecular Cancer*,* 18*,* 70; 30927908 10.1186/s12943-019-0994-2 PMC6441236

[56] Peng, D., Fu, M., Wang, M., Wei, Y., Wei, X. (2022). Targeting TGF-β signal transduction for fibrosis and cancer therapy. Molecular Cancer*,* 21*,* 104; 35461253 10.1186/s12943-022-01569-x PMC9033932

[57] Wen, S., Hou, Y., Fu, L., Xi, L., Yang, D. et al. (2019). Cancer-associated fibroblast (CAF)-derived IL32 promotes breast cancer cell invasion and metastasis via integrin beta3-p38 MAPK signalling. Cancer Letters*,* 442*,* 320–332; 30391782 10.1016/j.canlet.2018.10.015

[58] Paolillo, M., Schinelli, S. (2019). Extracellular matrix alterations in metastatic processes. International Journal of Molecular Sciences*,* 20*,* 4947; 31591367 10.3390/ijms20194947 PMC6802000

[59] Mhaidly, R., Mechta-Grigoriou, F. (2020). Fibroblast heterogeneity in tumor micro-environment: Role in immunosuppression and new therapies. Seminars in Immunology*,* 48*,* 101417; 33077325 10.1016/j.smim.2020.101417

