# Supplementary figures and images for: CRABP2 regulates infiltration of cancer-associated fibroblasts and immune response in melanoma
Source: Oncol Res. 2023 Dec 28;32(2):261–72. doi: 10.32604/or.2023.042345 (PMC10765133; doi:10.32604/or.2023.042345)

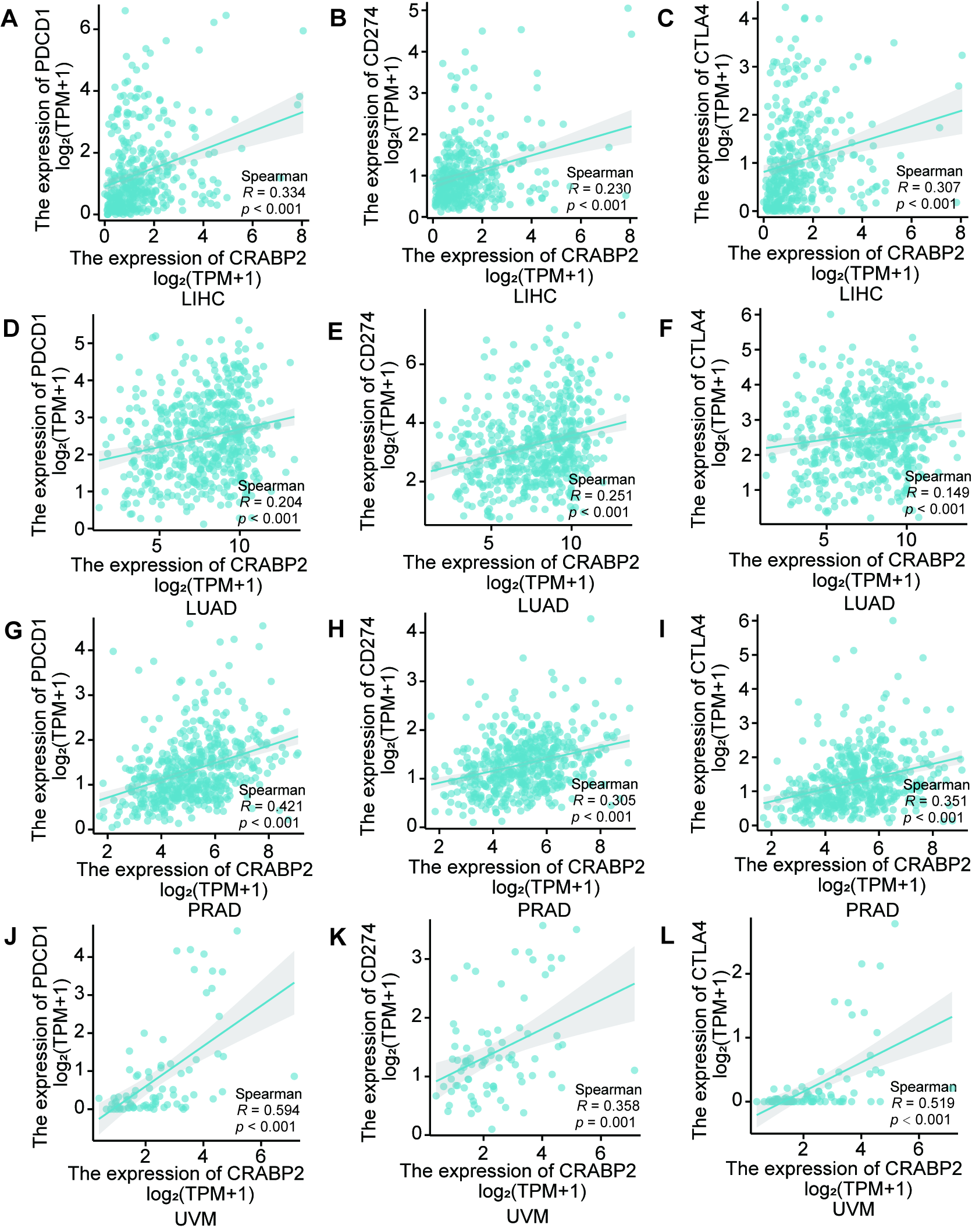

Supplement: Supplemental Figure S1 [file OncolRes-32-42345-s001.tif]

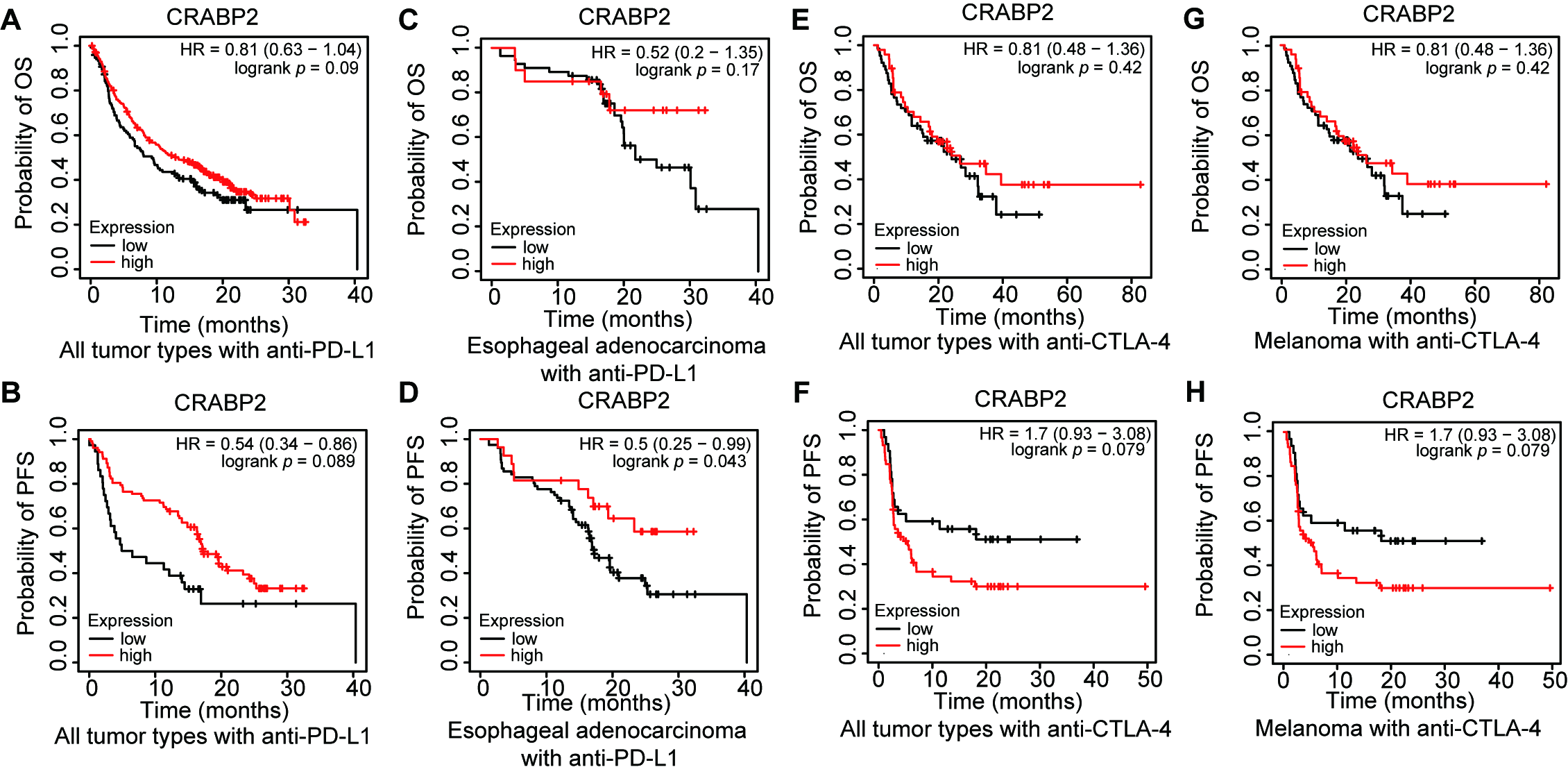

Supplement: Figure S2 [file OncolRes-32-42345-s002.tif]

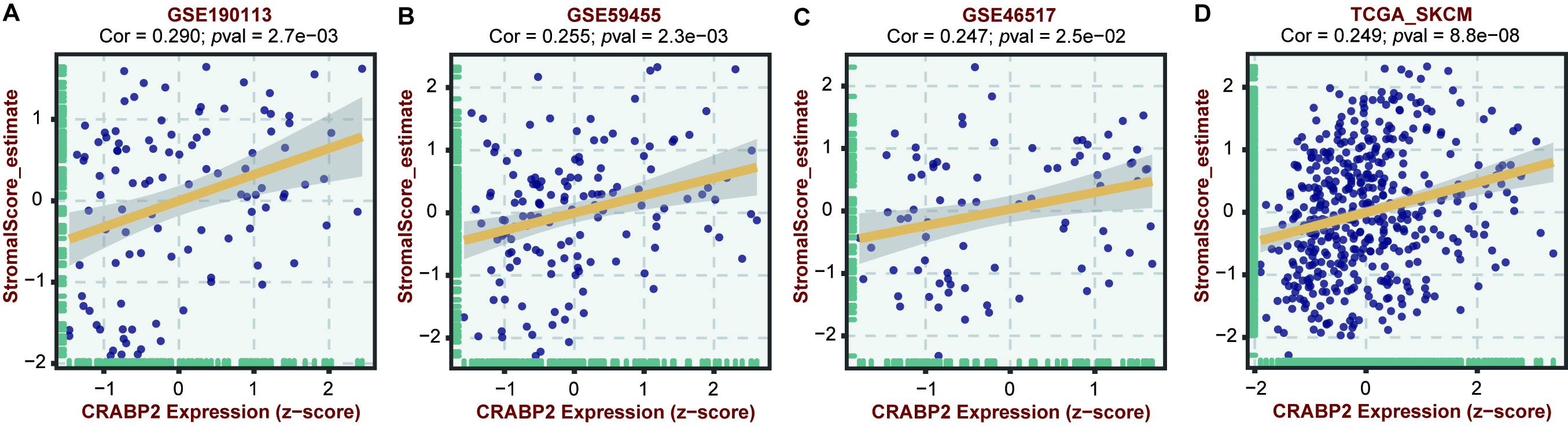

Supplement: Figure S3 [file OncolRes-32-42345-s003.tif]

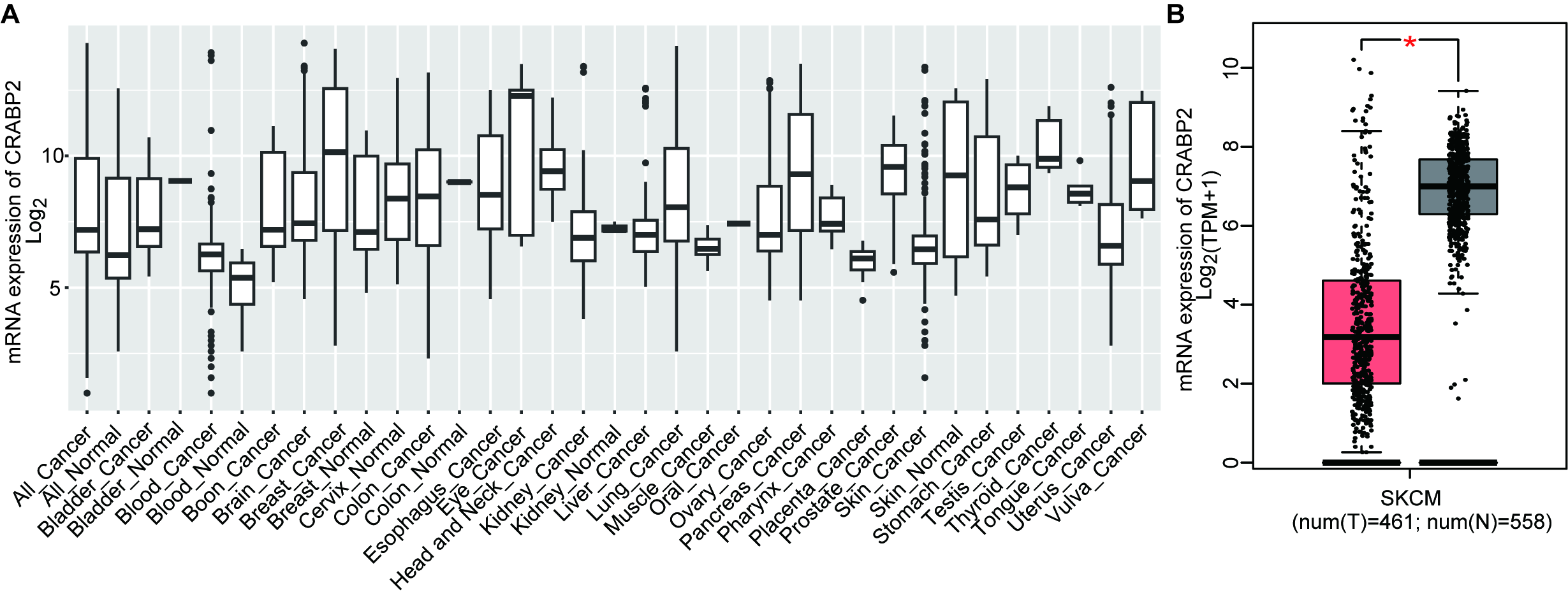

Supplement: Figure S4 [file OncolRes-32-42345-s004.tif]
